# Mechanical Properties of Crumb Rubber Mortar Containing Nano-Silica Using Response Surface Methodology

**DOI:** 10.3390/ma14195496

**Published:** 2021-09-23

**Authors:** Syafiqah Shahrul, Bashar S. Mohammed, M. M. A. Wahab, M. S. Liew

**Affiliations:** Civil and Environmental Engineering Department, Faculty of Engineering, Universiti Teknologi PETRONAS (UTP), Bandar Seri Iskandar 32610, Perak, Malaysia; syafiqah_18001894@utp.edu.my (S.S.); mubarakwahab@utp.edu.my (M.M.A.W.); shahir_liew@utp.edu.my (M.S.L.)

**Keywords:** crumb rubber, nano-silica, mortar, response surface methodology, scrap tires, recycling

## Abstract

Crumb rubber (CR) from scrap tires is used as a partial replacement of fine aggregates in cement paste. This promotes the sustainable development of the environment, economy, and society, as waste tires are non-biodegradable and flammable. They occupy large landfill areas and are breeding grounds for mosquitoes and rodents. Inclusion of CR in mortar leads to several improvements on the mixture properties such as ductility, toughness, and impact resistance. However, it exhibits lower strengths and Modulus of Elasticity (ME). Therefore, to promote the use of mortar containing CR, it is vital to improve its mechanical strength. Past studies proved that nano-silica (NS) improves the strength of concrete due to the physico-chemical effects of NS. This study aims to examine the mechanical properties of crumb rubber mortar containing nano-silica (NS-CRM) and to develop models to predict these properties using Response Surface Methodology (RSM). Two variables were considered, CR as partial replacement to sand by volume (0%, 7.5%, 15%), and NS as partial replacement to cement by weight (0%, 2.5%, 5%). The results demonstrated a significant improvement in the mechanical properties of CRM when incorporating NS, and the models developed using RSM were acceptable with a 2% to 3% variation.

## 1. Introduction

As the world’s population grows, so does the need for automobiles, especially in urban areas [1]. It is reported that five billion scrap tires are destined for landfills by 2030 [2]. Scrap tires are flammable and non-biodegradable, due to the presence of stabilizers, additives, and the cross-linked structure of the elastomeric polymer material [3], and they pose serious environmental, health, and aesthetic problems globally. They are bulky and require a large area in landfills. Due to their convex shape, they provide excellent breeding grounds for mosquitoes and rodents.

Reuse of scrap tires in construction will contribute to environment-friendly solutions for scrap tire disposal problems. Use of scrap tires in the form of crump rubber (CR) helps in improving the properties of the construction materials such as energy absorption [4], sound absorption [5,6], ductility [7], fatigue performance [8], impact resistance [9,10], electrical resistivity [11], heat resistivity [12], toughness [13], and post-crack strength [14]. Extensive research works conducted on concrete containing-CR as partial replacement to fine aggregate are well documented. However, research works on mortar containing CR are scarce and very limited.

Wongsa et al. [15] did a study on the mechanical properties of mortar by replacing sand with CR at 100% by volume. They reported that the density, thermal conductivity, compressive strength, and flexural strength of crumb rubber mortar (CRM) decrease as the percentage of CR increases. This is attributed to the weak bonding between CR and cement matrix, as well as the lower Modulus of Elasticity (ME) of the CR as compared to sand. However, a 100% replacement of sand by CR results in changing the failure mode of the mortar from brittle to ductile. It is suggested that CRM can be used for making environmentally friendly and lightweight bricks and blocks with improved thermal insulation. Yu and Zhu [16] investigated the properties of mortar by partially replacing sand with CR from 0% to 50% by volume. They concluded that the CRM exhibits reduction in ME, drying shrinkage, compressive strength, flexural strength, and splitting strength when the size of CR decreases, and the volume of CR increases. A strong relationship exists between the mechanical strengths and the porosity of the CRM. The mesopores volume of CRM increases as the percentage of CR increases. This is attributed to the hydrophobic nature of CR as it repels water and entraps air on its surface, which consequently increases the size of the pores in the hardened CRM. Angelin et al. [17] have studied the properties of mortar by partially replacing sand with CR from 0% to 30% by weight. The results indicated that the slump flow decreases as the percentage of CR increases. This reduction is caused by the increasing friction produced between the CR particles and cement paste. The density of CRM decreases with the increase in CR percentage, as the inclusion of CR causes the air content to increase. Due to the non-polar nature of CR particles, they are able to entrap air and repel water on its surface, leading to higher porosity and water absorption of CRM. Herrero et al. [18] have investigated the properties of CRM by partially replacing sand with CR at 30% to 60% by weight. Their findings have shown that the toughness, acoustic insulation, and thermal insulation increase as the percentage of CR increases. This is attributed to the elastic and energy dissipating characteristics of CR as a vibration-absorbing material.

Nanoparticles are used in concrete to enhance its performance and sustainability, such as compressive strength [19], flexural strength [20], tensile strength [21], ME [22], Poisson’s Ratio (PR) [23], electrical resistivity [24], cohesiveness [25], hydration rate [26], impermeability [21], and durability [27]. Nano-silica (NS) in concrete has two levels of effects which are the chemical effect and physical effect. In terms of chemical effect, through pozzolanic reaction, more calcium silicate hydrate (C-S-H) gel at final stages is formed as NS reacts with calcium hydroxide (Ca(OH)_2_), hence giving more strength, impermeability, and durability characteristics to the concrete [19]. While on the physical effect, it is able to decrease and densify the interfacial transition zone (ITZ) between the hardened cement matrix and aggregates due to its nano size. This leads to a lower porosity concrete with high performance [19]. In addition, when the nanoparticles are uniformly dispersed into the cement paste, they will act as a nucleus to tightly bond with cement hydrate and eventually enhance the hydration due to their large area-to-volume ratio [28]. Therefore, the objectives of this research work are to determine experimentally the mechanical properties of crumb rubber mortar containing nano-silica (NS-CRM) and to develop statistical models to predict these properties using Response Surface Methodology (RSM).

## 2. Materials and Methods

### 2.1. Materials Compositions and Properties

**Figure 1:**
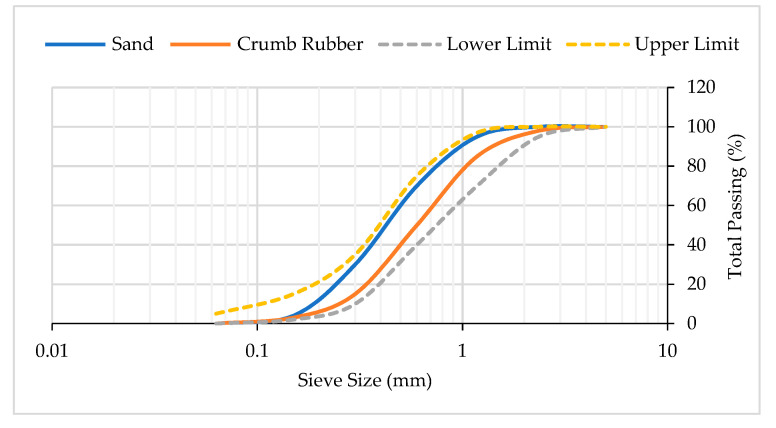
Particle size distribution graph of fine aggregates.

In this study, the cementitious materials used were ordinary Portland cement (OPC) and nano-silica (NS). Fine aggregates used were river sand and crumb rubber (CR). Included in the mixture were ordinary tap water and superplasticizer (SP). Type I Portland cement was used in the preparation of mortar mixes, and it meets the requirements of American Society for Testing Materials (ASTM) C150 [29]. NS used was in the form of white powder with electron grade standard, average particle sizes of 10 nm to 25 nm and density of 0.15 g/cm^3^. The NS was used to partially replace 0%, 2.5%, and 5% of cement by weight. Substituting a high percentage of NS will cause a further reduction in strengths, and it is uneconomical [30]. The chemical compositions and the physical properties of cement and NS are presented in Table 1 and Table 2 below. River sand with particle sizes of 0.3 mm to 1.18 mm was used and it meets the requirements of ASTM C144-18 [31]. CR with particle sizes of 0.3 mm to 2.36 mm was used to partially replace 0%, 7.5%, and 15% of river sand by volume. It was determined from the available literature that using a high percentage of CR replacement will lead to a dramatic reduction in the strengths of CRM [17,32]. Table 3 shows the physical properties of river sand and CR used. Figure 1 illustrates the particle size distribution (PSD) graph of fine aggregates used. Ordinary tap water available in the laboratory was used and the quality conforms to the requirement of ASTM C1602/C1602M-18 [33]. Aqueous solution of modified polycarboxylates under the brand name of Sika Viscocrete-2044 was used in the mortar mixes. Table 4 shows the chemical compositions of SP used.

### 2.2. Mix Proportions

For all mixes, the water to cementitious materials (w/c) ratio and sand to cementitious materials (s/c) ratio were kept constant at 0.35 and 1.5, respectively. In order to study the mechanical properties of NS-CRM, thirteen mixes were prepared using Response Surface Methodology (RSM) with different ratios of NS (0%, 2.5%, 5%) as partial replacement to cement by weight, while CR was replaced at varying percentages (0%, 7.5%, 15%) by volume of river sand. Each mixture was given a distinctive name as shown in Table 5. For instance, mixture CR7.5-NS2.5 was a mortar mixture with 7.5% CR and 2.5% NS. There were five mix designs which contained the same percentage of CR and NS. These repetitive designs produced by RSM were to ensure they measured the pure error and stabilized the variance of the predicted responses.

### 2.3. Response Surface Methodology

Response Surface Methodology (RSM) discovers the relationships between one or more response variables [34]. RSM uses a series of designed experiments to achieve an ideal response. It is a specialized statistical method which is most commonly used for simulating processes and experiments [35]. RSM is implemented using Design-Expert 10 software where runs are known as a trial of progression tests and the output response depends solely on the input data variables. For this study, Central Composite Design (CCD) was chosen as the design tool. CCD is an effective design choice that is suitable for sequential experimentation [36]. It allows a reasonable amount of information for testing the lack of fit where it does not involve an unusually large number of design points [37].

In this study, two variables were used as partial substitutes, which were CR and NS. Sand was replaced by CR in varying volumes of 0%, 7.5% and 15%. Cement was replaced by NS in varying weight of 0%, 7.5%, and 15%. Thirteen mixes were established by the RSM software, using various combinations of the variables as illustrated in Table 5. For each mix, the amount of water used was kept constant. The 28 days compressive strength, flexural tensile strength, direct tensile strength, drying shrinkage, Modulus of Elasticity (ME), and Poisson’s Ratio (PR) of each of the mixes was determined in the laboratory and was considered as the responses for the RSM analysis and mixtures optimization.

### 2.4. Sample Preparations and Test Methods

Thirteen design mixes of mortar were recommended by the CCD tool and each design mix contained three specimens with three replicates that were cast, cured, and tested. The first step involved the preparation of materials and specimens. At this stage, preparation of molds, mixing of NS with ordinary tap water, mixing of SP with ordinary tap water, and sieving of fine aggregates were performed. Next, cement, river sand, and CR were dry mixed for about 3 to 5 min. Then NS, water, and SP were added, and the mixing continued for about 5 to 7 min. Finally, the specimens were cast and demolded after 24 h. They were kept in the laboratory curing tank with a temperature of 20 °C ± 2 °C until they were ready to be tested. To observe changes in strengths of each mix, testing of specimens took place on day 7, 14, and 28.

After all specimens were tested, the results were then input into the CCD. Each response was then analyzed to produce the Analysis of Variance (ANOVA), diagnostics, and model graphs. Next, numerical optimization was done by setting the goals for each response. Once the goals were set up, the software generated a few optimal conditions (optimized mix proportions) for each response that need to be verified by conducting laboratory work. These new optimized mix proportions were then cast, cured, and tested. The results for each test were recorded. Lastly, the percentage error between the experimental and theoretical strengths was calculated. Table 6 shows the details of specimens used, size of molds, test conditions, and the ASTM standards for the different types of tests.

## 3. Results and Discussion

### 3.1. Effect of Crumb Rubber and Nano-Silica on Hardened Properties of NS-CRM

#### 3.1.1. Compressive Strength

**Figure 2:**
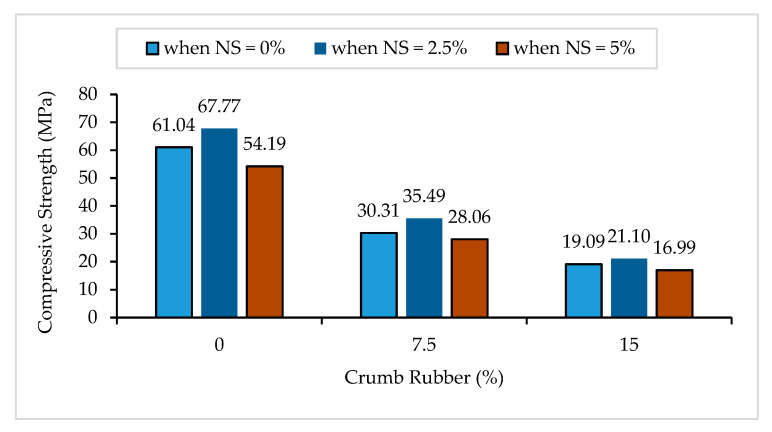
Compressive strength of NS-CRM mixes at 28 days.

The compressive strength results of crumb rubber mortar containing nano-silica (NS-CRM) are presented in Figure 2. A considerable decrease in the compressive strengths was observed with an increasing percentage use of crumb rubber (CR). The strengths decreased by 50.3% and 68.7% when incorporating 7.5% and 15% of CR at 0% of NS. This trend is expected due to the hydrophobic nature of CR which repulses water and traps air on its surface, leading to weak bonding between the cement matrix and CR particles. The poor bonding leads to the thickening of the interfacial transition zone (ITZ) and the lowering of stress transfer. Furthermore, the trapped air on the CR surface becomes void and increases the porosity of the hardened mortar, consequently leading to a lower strength.

It was observed that the increase in NS from 0% to 2.5% had a good influence on the compressive strengths of mortar. The strengths increased by 11.0%, 17.1%, and 10.5% between samples having 0% and 2.5% of NS content at 0%, 7.5%, and 15% of CR replacement, respectively. The positive effect of NS on the compressive strengths is attributed to its physico-chemical effect within the mortar. In a secondary hydration reaction, the NS reacts with calcium hydroxide (Ca(OH)_2_) liberated from cement hydration to form more calcium silicate hydrate (C-S-H) gel [47]. This is known as a pozzolanic reaction. The C-S-H produced from the cement hydration and the pozzolanic reaction is responsible for the strength of the mortar [27]. On the physical effect, the nano-sized particles of the NS fill up the voids within the matrix, thereby densifying and refining the pore structure of the mortar [28,48]. However, at a higher NS percentage of 5%, a sudden drop in compressive strengths were observed for all CR replacements. This is due to the excessive content of NS which acts as a nano-aggregate within the CRM and leads to a less dense microstructure [20].

**Figure 3:**
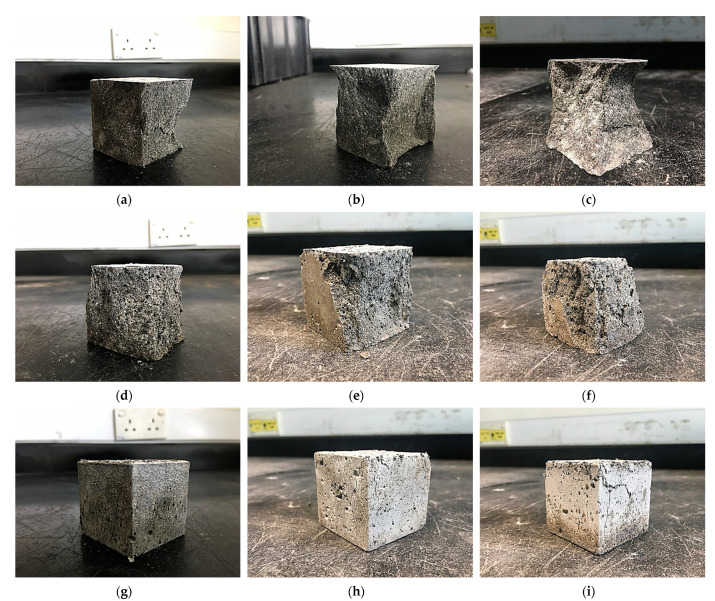
Crushed cube samples of NS-CRM mixes at 28 days: (**a**) CR0-NS0; (**b**) CR0-NS2.5; (**c**) CR0-NS5; (**d**) CR7.5-NS0; (**e**) CR7.5-NS2.5; (**f**) CR7.5-NS5; (**g**) CR15-NS0; (**h**) CR15-NS2.5; (**i**) CR15-NS5.

The failure mode of the NS-CRM is influenced by the CR and NS content. At 0% CR and NS, the NS-CRM experienced brittle failure mode as shown Figure 3a–c. The severity of the failure increased at higher NS percentages. This is due to the increase in toughness of the mortar by the NS. However, as the CR replacement increased, the failure mode became less severe as illustrated in Figure 3d–i for 7.5% and 15% of CR replacements, respectively. This is attributed to the enhanced energy absorption capacity of the NS-CRM with increasing CR percentage. As reported by Yilmaz and Degirmenci [49], the addition of CR particles having low stiffness increase the flexibility of the CRM, allowing it to absorb more energy and exhibit a lower number of cracks at failure despite having low strength.

#### 3.1.2. Flexural Tensile Strength

**Figure 4:**
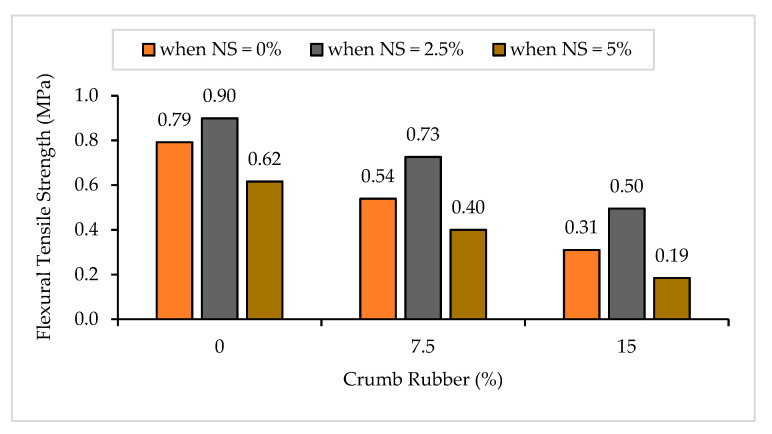
Flexural tensile strength of NS-CRM mixes at 28 days.

**Figure 5:**
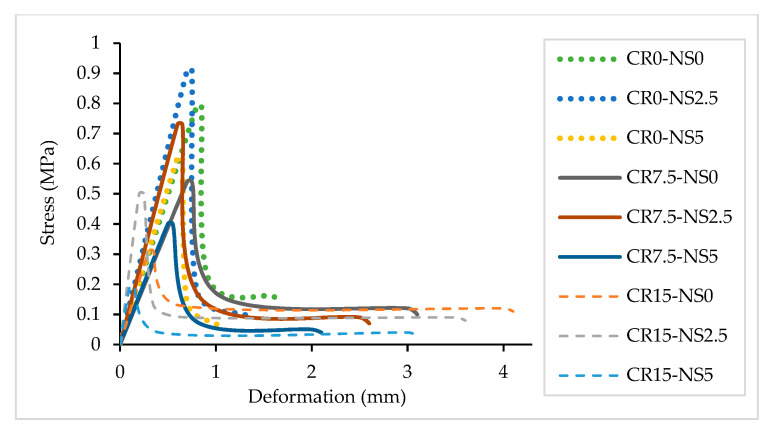
Deformation of NS-CRM samples at 28 days.

The flexural tensile strength results of NS-CRM are illustrated in Figure 4. An enormous decrease in the flexural tensile strengths was observed with an increasing percentage use of CR. The strengths decreased by 31.9% and 60.9% when incorporating 7.5% and 15% CR at 0% NS. The declining trend in the strengths of mortar samples is primarily due to the hydrophobic characteristic of CR that leads to a higher void content in the mortar. It allows stress concentration across the pore to happen, thus causing formation of micro-cracks [50]. Besides, CR is not compatible with sand. It has lower strength, stiffness, specific gravity, and load-carrying capacity, leading to a reduction in strength when replaced with a portion of sand [49]. Moreover, the presence of CR leads to a more porous hardened mortar due to the change of aggregate grading in the mortar mix from continuous to a non-continuous grading. Hence, it causes more cavities to form due to the inability of the voids in the aggregate to be filled by the CR particles [51]. As the percentage of CR was increased, the mortar samples behavior substantially changed from brittle to ductile when the peak strength was reached. This is due to the elastic properties [52] and better energy absorption [4] of CR. Figure 5 shows that the mortar samples containing higher percentage of CR were able to withstand the load for a longer period of time before they broke into two, regardless of them having low flexural tensile strength.

In contrast, it was observed that the increase in NS from 0% to 2.5% had a beneficial impact on the flexural tensile strengths. The strengths increased by 13.5%, 34.7%, and 59.7% between samples having 0% and 2.5% of NS content at 0%, 7.5%, and 15% of CR replacement, respectively. The positive effect of NS on the flexural tensile strengths is attributed to the physical properties of NS which acts as a filler in filling the nano-voids between aggregates and cement matrix [53]. Supported by Li et al. [28], NS has the capability to improve the strength, toughness, and stiffness of mortar due to its large area-to-volume ratio, therefore it acts as a physical filler in filling the nano-voids between cement and aggregates. However, when 5% of NS was incorporated into the mixture, an enormous drop in flexural tensile strength was observed for all CR replacements. This is due to the high percentage of NS which promotes the packing of particles, decreasing the volume between them and decreasing the free water in the mixture, hence causing a higher internal friction between solid particles [54].

#### 3.1.3. Direct Tensile Strength

**Figure 6:**
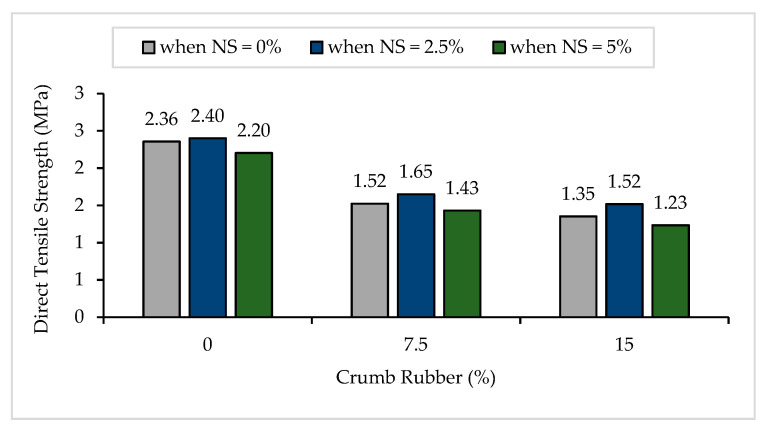
Direct tensile strength of NS-CRM mixes at 28 days.

**Figure 7:**
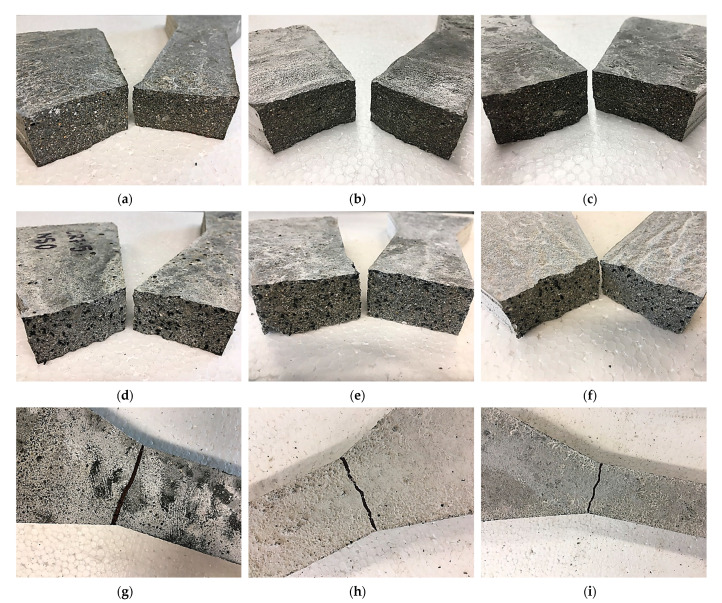
Tested dog-bone samples of NS-CRM mixes at 28 days: (**a**) CR0-NS0; (**b**) CR0-NS2.5; (**c**) CR0-NS5; (**d**) CR7.5-NS0; (**e**) CR7.5-NS2.5; (**f**) CR7.5-NS5; (**g**) CR15-NS0; (**h**) CR15-NS2.5; (**i**) CR15-NS5.

The direct tensile strength results of NS-CRM are presented in Figure 6. A general decline in the direct tensile strengths was observed with increasing percentage use of CR. The strengths decreased by 35.3% and 42.6% when incorporating 7.5% and 15% of CR at 0% of NS. The declining trend in the strengths of mortar samples is caused by the presence of air content in the mixture, due to the non-polar nature of CR particle which has the tendency to entrap air on its rough surface. Therefore, it may attract air because it has the propensity to repel water. It was observed that the crack failure occurred at a point of abrupt change in the cross-sectional area due to the high-stress level, as illustrated in Figure 7. Although the samples with 15% of CR achieved low direct tensile strength, they remained intact even after failure. This indicates that the post-crack strength of the samples improves with the presence of CR particles. Supported by Yilmaz and Degirmenci [49], the control mortar samples split into two pieces immediately after cracking, whereas samples containing CR particles showed a larger deformation without complete disintegration.

Conversely, it was observed that the increase in NS from 0% to 2.5% had a positive influence on the direct tensile strength. The strengths increased by 1.9%, 8.3%, and 12.1% between samples having 0% and 2.5% of NS content at 0%, 7.5%, and 15% of CR replacement, respectively. NS enhances the durability, as well as the strength of the mortar mixture because the ultra-fine particles fill the voids of C-S-H structure and provide a more homogenous distribution of hydrated products. Furthermore, NS increases the density [55], reduces porosity, and improves the bond between aggregates and cement matrix [48]. However, it was observed that incorporating 5% of NS into the mixture had caused a slight drop in direct tensile strength for all CR replacements. According to Sobolev et al. [56], a higher percentage of NS replacing cement in the mixture does not lead to an improvement in the strength because of the improper dispersion of NS particles. Due to their high surface energy, the tendency towards agglomeration is high, thus leading to a weak zone created between the cement matrix and aggregates in the mortar. Therefore, it is vital to attain the composite materials with improved properties.

#### 3.1.4. Drying Shrinkage

**Figure 8:**
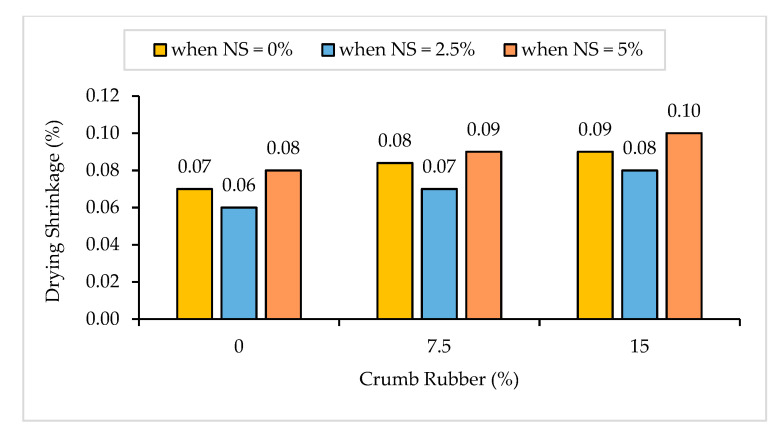
Drying shrinkage of NS-CRM mixes at 28 days.

The drying shrinkage results of NS-CRM are illustrated in Figure 8. A steady incline in the shrinkage results was observed with an increasing percentage use of CR. The shrinkage results increased by 14.3% and 28.6% when incorporating 7.5% and 15% of CR at 0% of NS. According to Yu and Zhu [16], the CRM appears to depend on the size and content of CR. Clearly, as the CR percentage used is high, the drying shrinkage will be high too. The inclining trend in the shrinkage of mortar samples is because the CR causes an increment of air content and voids in the mortar mixture.

On the contrary, it was observed that the increase in NS from 0% to 2.5% had a positive influence on the drying shrinkage results. The shrinkage results decreased by 14.3%, 12.5%, and 11.1% between samples having 0% and 2.5% of NS content at 0%, 7.5%, and 15% of CR replacement, respectively. Arzadnia et al. [57] have reported that the reduction in drying shrinkage of the hardened samples is due to the reduction in permeability of the mixture. The C-S-H produced from pozzolanic reaction of NS allows it to fill the capillary pores and restrain the loss of moisture in the pores, causing the drying shrinkage of the hardened samples to decrease. However, as the percentage of NS incorporated increased to 5%, a substantial increase in NS-CRM drying shrinkage results were observed. Neto et al. [58] have stated that high replacement of cement by NS causes the alteration of pore size and increase in mesopores volume due to agglomeration of unreacted NS which leads to excessive self-desiccation.

#### 3.1.5. Modulus of Elasticity

**Figure 9:**
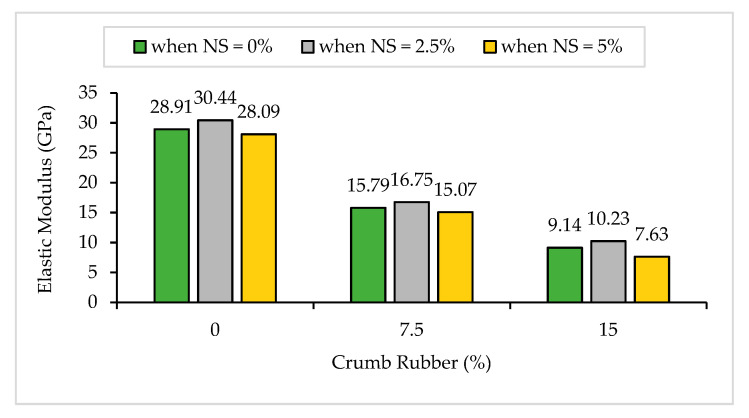
Modulus of Elasticity of NS-CRM mixes at 28 days.

**Figure 10:**
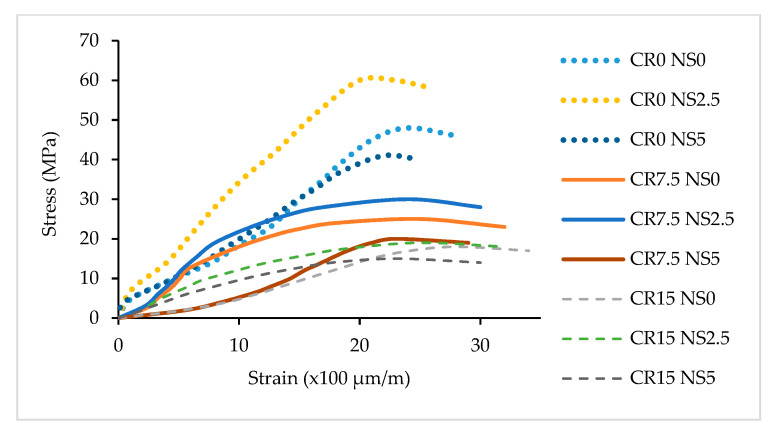
Stress-strain of NS-CRM mixes at 28 days.

The Modulus of Elasticity (ME) results of NS-CRM are presented in Figure 9. A dramatic fall in the ME results was observed with increasing percentage use of CR. The ME decreased by 45.4% and 68.4% when incorporating 7.5% and 15% of CR at 0% of NS. The sharp downward trend in the ME results is due to the physical properties of CR particles which are less stiff as compared to the hardened cement matrix in the mortar [49]. Therefore, it allows the CR to act as tiny springs inside the hardened mortar. In Figure 10, it was observed that the peak stress achieved by samples having 15% of CR content at 0%, 2.5%, and 5% of NS replacement were 15 MPa, 18 MPa, and 19 MPa. Even so, the strain achieved was the highest due to the elastic properties of CR.

However, it was observed that the increase in NS from 0% to 2.5% had a positive influence on the ME results. The ME increased by 5.3%, 6.1%, and 11.9% between samples having 0% and 2.5% of NS content at 0%, 7.5%, and 15% of CR replacement, respectively. According to Aleem et al. [30], the improvement of strength is due to the behavior of NS that acts as a filler to enhance the microstructure, as an activator to encourage pozzolanic reaction, along with acts as a nucleation effect with Ca(OH)_2_ to perform pozzolanic effects. Hence, it leads to more accumulation and precipitation of the hydrated products in the pores that are initially filled with water. Consequently, it leads to the formation of dense, compact, and homogeneous microstructure, and better stiffness of CRM. However, it was observed that incorporating 5% of NS into the mixture had caused a marginal drop in the ME for all CR replacements. Li et al. [59] have concluded that NS cannot be uniformly dispersed when a large quantity of it is used due to its great surface energy. Therefore, the unreacted NS causes agglomeration to occur and affects the microstructure of the CRM.

#### 3.1.6. Poisson’s Ratio

**Figure 11:**
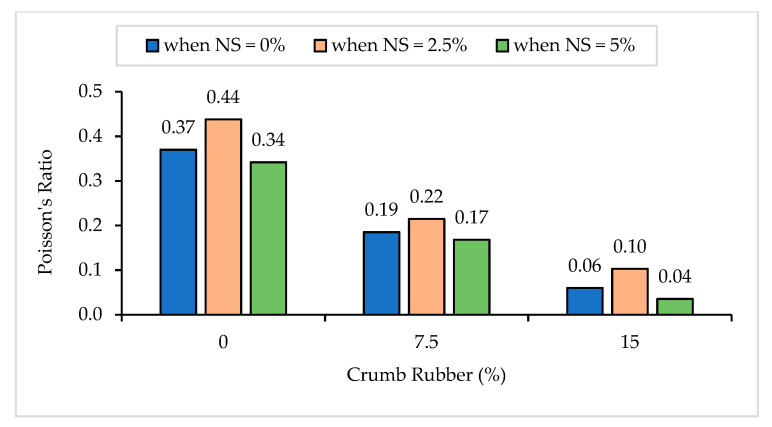
Poisson’s Ratio of NS-CRM mixes at 28 days.

The Poisson’s Ratio (PR) results of NS-CRM are illustrated in Figure 11. A gradual decline in the PR results was observed with increasing percentage use of CR. The PR decreased by 50.0% and 83.8% when incorporating 7.5% and 15% of CR at 0% of NS. The declining trend in the PR results is due to the presence of CR which is less stiff as compared to the hardened cement matrix in the mortar [49]. Due to the elastic properties of CR [52], it acts as a tiny spring in the hardened mortar which leads to an increase in the deformability of the CRM and consequently decreasing the PR. Sideris et al. [60] have implied that there is a linear relationship between ME, PR, and compressive strength of mortar. Sideris [61] added that the linear relationship between compressive strength of mortar leads to the possibility of estimating the ME and PR. Therefore, incorporating a high percentage of CR leads to lower compressive strength due to the non-polar nature of CR. Subsequently, decreases the ME due to the fact that there is a concentration of stress at the aggregate–mortar interface due to the large difference in the ME between the aggregate and the mortar [62] and hence, causing the PR to decrease as well.

Nonetheless, it was observed that the increase in NS from 0% to 2.5% had a positive impact on the PR. The PR increased by 18.4%, 16.2%, and 71.7% between samples having 0% and 2.5% of NS content at 0%, 7.5%, and 15% of CR replacement, respectively. Mukharjee et al. [63] have stated that the physical effect is due to the nano-size of NS particles which are able to fill up the nano-sized voids of the ITZ. The chemical effect is due to the physico-chemical effect in which NS reacts with the Ca(OH)_2_ and generates more C-S-H gel. Thus, this leads to the densification of the ITZ and consequently decreases its thickness. However, it was observed that incorporating 5% of NS into the mixture had caused a marginal drop in the PR for all CR replacements. This happens due to the high percentage of NS incorporated, which leads to the agglomeration of unreacted NS.

### 3.2. Mathematical and Statistical Models Using Response Surface Methodology

#### 3.2.1. Response Surface Methodology Modeling

The experimental mix designs and the results are presented in Table 7 below. For each mix design, three samples with three replicates were prepared. Thus, the results presented in the table below were the average of three samples tested.

#### 3.2.2. Statistical Models and Analysis of Variance

A summary of the Analysis of Variance (ANOVA) for the quadratic response model is presented in Table 8. The F-values of the models were 113.91, 138.81, 285.63, 102.13, 765.52, and 73.58 for compressive strength, flexural tensile strength, direct tensile strength, drying shrinkage, ME, and PR, respectively. This indicated that the models were all significant. There was only 0.01% possibility that a model F-value of the respective magnitude could occur due to noise for all responses. Besides, the significance of all models and all their terms were verified using the 95% confidence interval (*p* < 0.05), as depicted in Table 8.

The equations, in terms of coded factors, could be used to make predictions about the responses for the given levels of each factor. The high levels of the factors were coded as +1 and the low levels of the factors were coded as −1. These equations were useful in detecting the relative impact of the factors by comparing the factor coefficients. The models’ equation for predicting the mechanical strengths on the basis of variables are shown below, where ‘*A*’ and ‘*B*’ indicate the CR and NS in percentages, respectively.

Compressive Strength (MPa) = +36.24 − 20.97(*A*) − 1.87(*B*) + 1.19(*AB*) + 8.38(*A*^2^) − 6.87(*B*^2^) (1)

Flexural Tensile Strength (MPa) = +0.73 − 0.22(*A*) − 0.073(*B*) + 0.013(*AB*) − 0.015(*A*^2^) − 0.24(*B*^2^) (2)

Direct Tensile Strength (MPa) = +1.65 − 0.48(*A*) − 0.016(*B*) + 8.25 × 10^−3^(*AB*) + 0.31(*A*^2^) − 0.17(*B*^2^) (3)

Drying Shrinkage (%) = +0.071 + 0.010(*A*) + 4.33 × 10^−3^(*B*) + 0(*AB*) − 1.17 × 10^−3^(*A*^2^) + 0.016(*B*^2^) (4)

Modulus of Elasticity (GPa) = +16.83 − 10.07(*A*) − 0.51(*B*) − 0.17(*AB*) + 3.31(*A*^2^) − 1.60(*B*^2^) (5)

Poisson’s Ratio = +0.22 − 0.16(*A*) − 0.012(*B*) + 8.75 × 10^−4^(*AB*) + 0.042(*A*^2^) − 0.052(*B*^2^) (6)

Referring to Table 9, Pred R^2^ is a measure of how well the model predicts a response value, whereas Adj R^2^ is for the number of parameters in the model relative to the number of points in the design. It is a measure of the amount of variation about the mean explained by the model. The Adj R^2^ and Pred R^2^ should be within approximately 0.20 of each other to be in reasonable agreement. If they are not, there may be a problem with either the data or the model. In this study, all responses were in good agreement. The inconsistency of the models with reference to the experimental data were checked using their SD values. The experimental data fitted the developed models with a higher degree of correlation because the SD values were lower as compared to the µ of the models. Besides, AP is a measure of the adequate signal. As they are greater than 4, the developed models can be used for navigating the design space.

**Figure 12:**
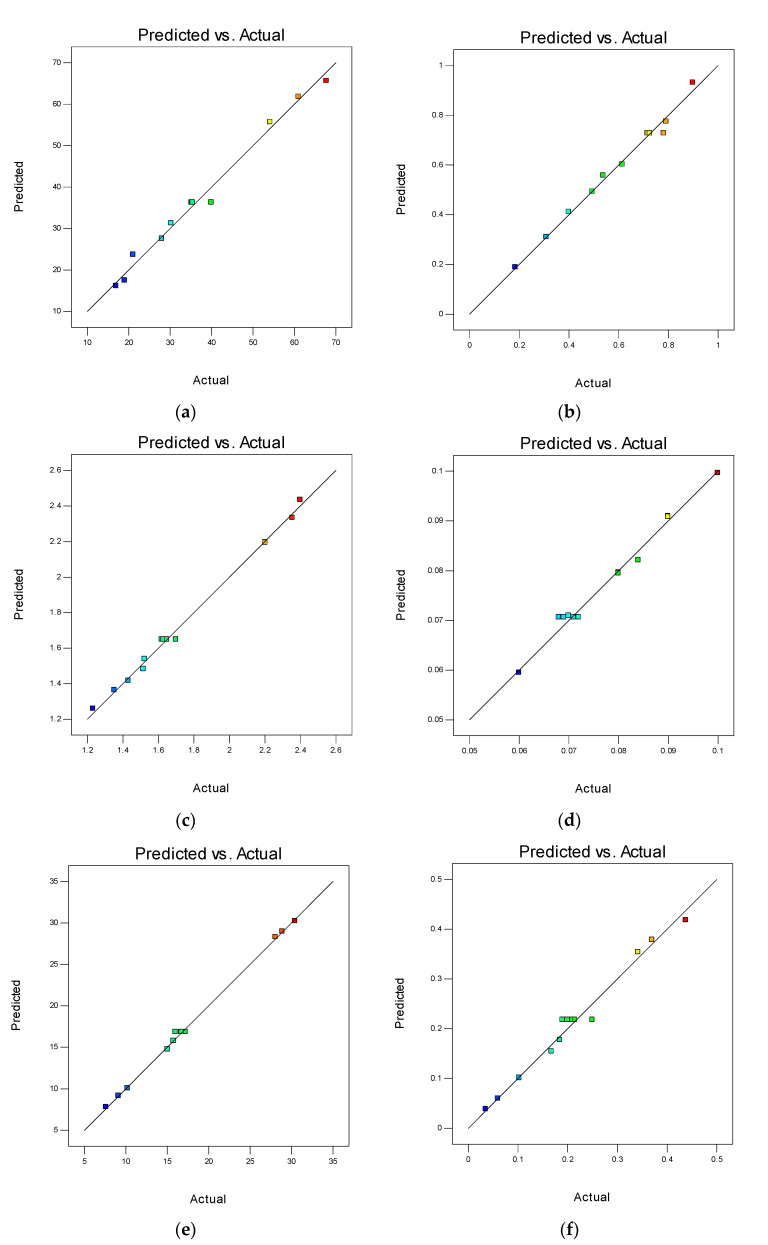
Predicted vs. actual plots for developed response models: (**a**) Compressive Strength; (**b**) Flexural Tensile Strength; (**c**) Direct Tensile Strength; (**d**) Drying Shrinkage; (**e**) Modulus of Elasticity; (**f**) Poisson’s Ratio.

The diagnostics of the predicted response against the actual response values are shown in Figure 12. This type of graph helps to detect observations that are not well predicted by the model and the data points should be split evenly by 45°. As illustrated in Figure 12a–f, the experimental data were in good agreement and fitted to the predicted results from the models. Therefore, the developed response models were relevant and suitable for predicting the mechanical strengths and models of NS-CRM.

**Figure 13:**
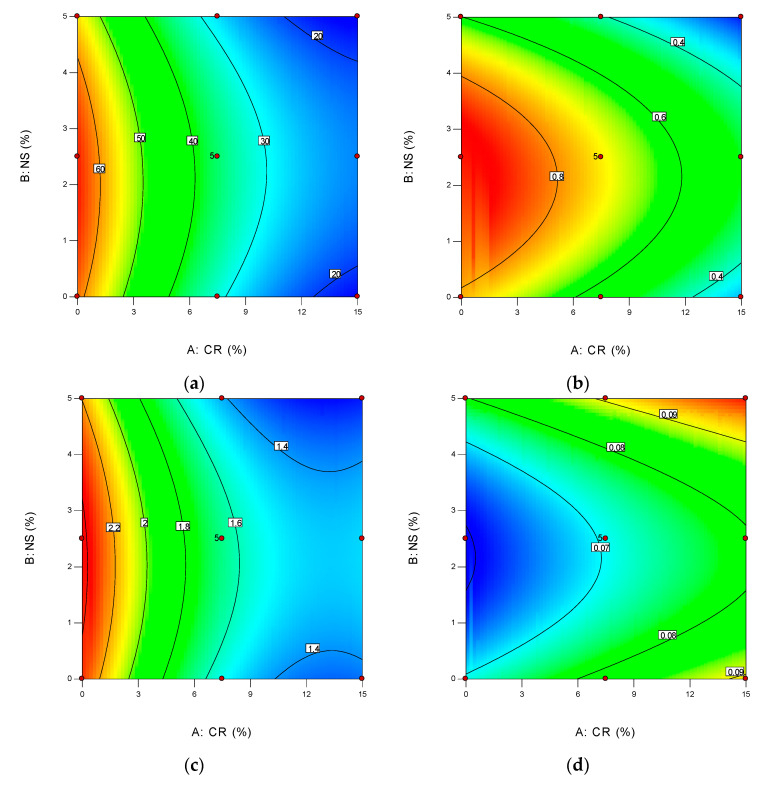

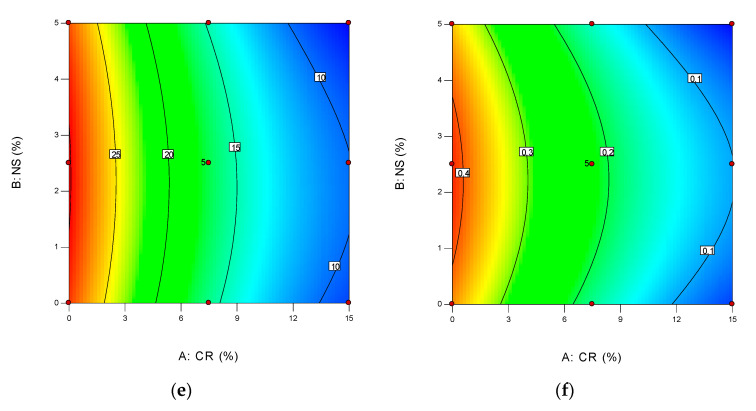
2D contour plots for developed response models: (**a**) Compressive Strength; (**b**) Flexural Tensile Strength; (**c**) Direct Tensile Strength; (**d**) Drying Shrinkage; (**e**) Modulus of Elasticity; (**f**) Poisson’s Ratio.

A contour plot is a 2-dimensional (2D) representation of the response plotted against combinations of numeric factors and/or mixture components, as illustrated in Figure 13. It is able to illustrates the relationship between the responses, mixture components and/or numeric factors. Good interaction between variables and responses can be determined when the contour lines are oval in shape. As illustrated below, there were a perfect interaction between CR and NS for all the responses, with flexural tensile strength and drying shrinkage responses had the best interaction as shown in Figure 13b,d. The reddish areas on the contour plots showed the best combinations to give the optimum values, while the bluish area on the plot for drying shrinkage demonstrated the best.

**Figure 14:**
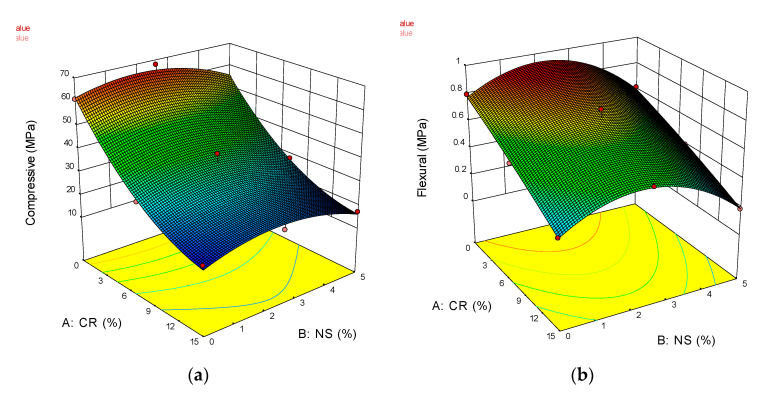

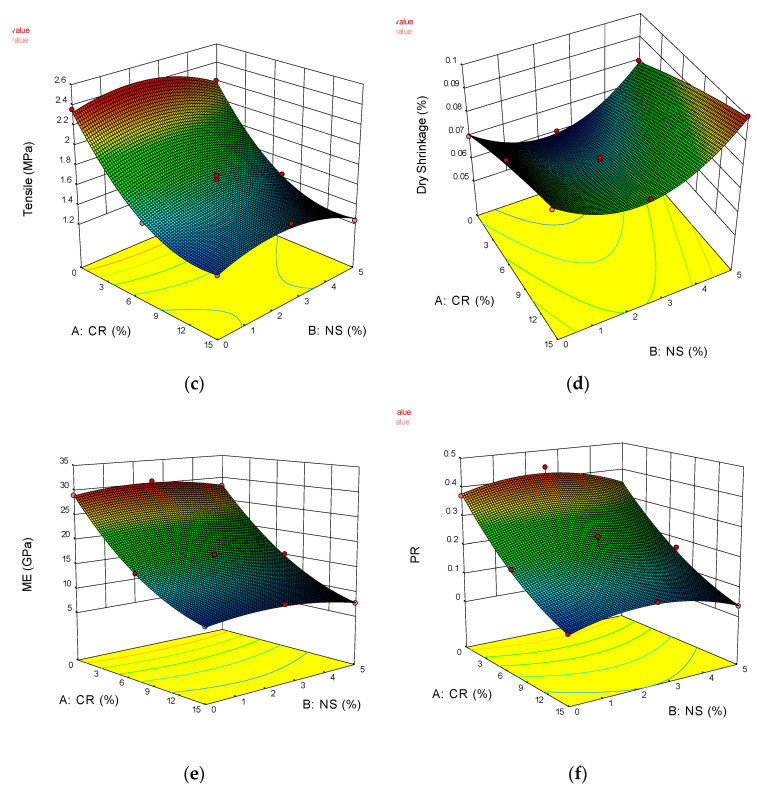
3D contour plots for developed response models: (**a**) Compressive Strength; (**b**) Flexural Tensile Strength; (**c**) Direct Tensile Strength; (**d**) Drying Shrinkage; (**e**) Modulus of Elasticity; (**f**) Poisson’s Ratio.

A 3-dimensional (3D) surface plot is a projection of the contour plot giving shape in addition to color and contour, as illustrated in Figure 14. It was observed that CR exhibited higher and negative effects on most of the responses as compared to NS.

#### 3.2.3. Optimization and Experimental Validation

Multi-objective optimization was carried out using the Central Composite Design (CCD) module in the Response Surface Methodology (RSM) tool to determine the optimum mixtures of NS-CRM that could produce the targeted compressive strength inputs, which were 20 MPa, 35 MPa, and 50 MPa. The variables and other responses were targeted to be in the range of lower and upper limits to ensure the CCD tool captured all possible combinations of results. The multi-objective optimization criteria are summarized in Table 10.

**Figure 15:**
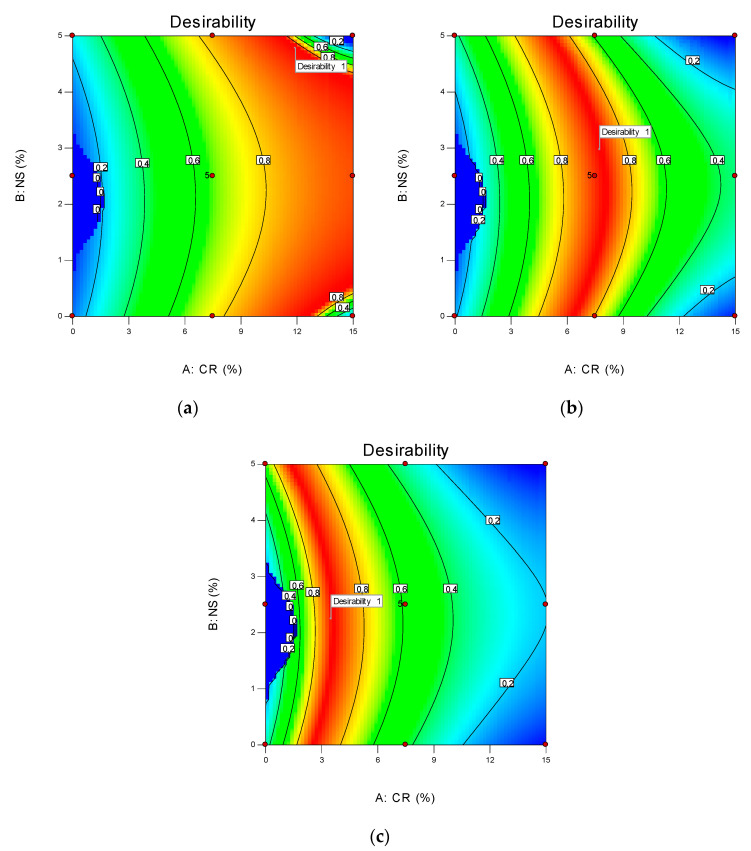
Desirable compressive strength of NS-CRM: (**a**) 20 MPa; (**b**) 35 MPa; (**c**) 50 MPa.

The results for the multi-objective optimization are shown in Table 11. Based on the optimization goals, the best mixture proportions selected by the CCD tool in RSM software were obtained by incorporating 12%, 8%, and 3% of CR as a fine aggregate replacement and 5%, 3%, and 2% of NS as replacement to cement to achieve 20 MPa, 35 MPa, and 50 MPa in compressive strength, with combined desirability of 100% as shown in Table 11 and Figure 15.

An experimental validation study was carried out using the optimized mix proportions produced by CCD tool in RSM in order to validate the optimization results and the overall response models. The compressive strength, flexural tensile strength, direct tensile strength, drying shrinkage, ME, and PR were then tested experimentally after 28 days of curing, and the results were compared with the predicted values given by CCD tool in the RSM. The percentage errors between the predicted responses and the experimental results were calculated using Equation (7) below. A summary of the experimental and predicted results is illustrated in Table 11. The experimental and the predicted values were in good agreement with one another, with calculated errors of 2% to 3% for all responses of NS-CRM.

(7) $$\mathrm{Error}\;(\%)=|\frac{Experimental-Predicted}{Experimental}|\;\times \;100$$

### 3.3. Microstructural Analysis of NS-CRM

#### 3.3.1. Scanning Electron Microscope

Scanning Electron Microscope (SEM) image analysis can reveal detailed size, shape, and structural nanomaterial information because it can achieve a resolution better than 1 nm. For this study, three crushed samples were sent to the laboratory for testing, which included CR7.5-NS0, CR7.5-NS2.5, and CR7.5-NS5. In order to prove that NS could act as a filler in filling the voids created between the cement matrix and CR, the samples provided had the same percentage of CR but varied percentages of NS.

1 1. CR7.5-NS0

**Figure 16:**
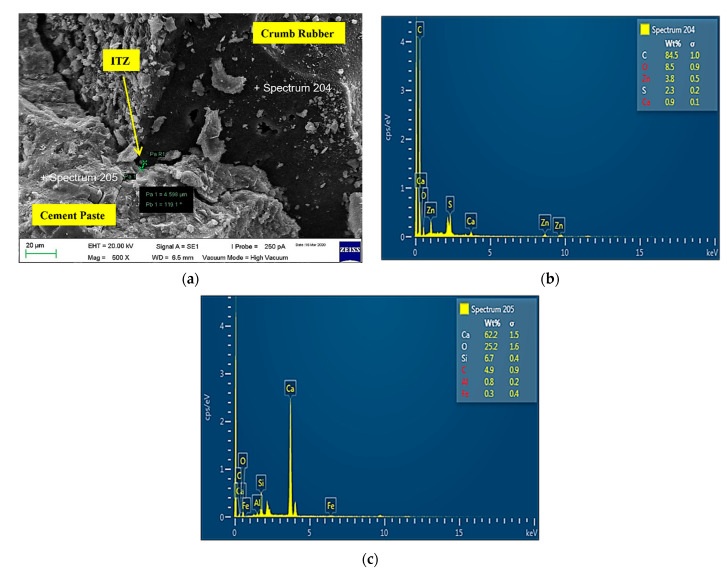
CR7.5-NS0 sample: (**a**) Microstructure ITZ = 4.598 µm; (**b**) EDX of crumb rubber; (**c**) EDX of cement.

Figure 16 shows the microstructure and Energy Dispersive X-Ray Spectroscopy (EDX) images of sample CR7.5-NS0. Based on the image analysis produced, ITZ created between the cement matrix and CR was 4.598 µm. It confirmed that the mixture containing only CR had higher air content due to the non-polar nature of CR particles. Mohammed et al. [64] stated that CR has the tendency to repel water and entrap air on its rough surface, hence causing ITZ to be created. This causes the bonding between CR and cement paste formed in hardened mortar to be weakened due to less adhesion of the mortar matrix. According to Hunag et al. [65], the interface gaps between CR and grout can be seen clearly because CR is a type of organic high molecular weight material. The bonding between material interfaces is weak when it is added into the cement mixture. Therefore, the material interfaces are likely to be damaged when the specimen is under an external force. Supported by Su et al. [66], the failure of samples is primarily caused by debonding between the aggregates and the cement paste. Incorporating CR particles results in a weaker bond between the CR and the surrounding cement paste. It is proven that the adhesion between the CR particles and the cement matrix was poor.

2 CR7.5-NS2.5

**Figure 17:**
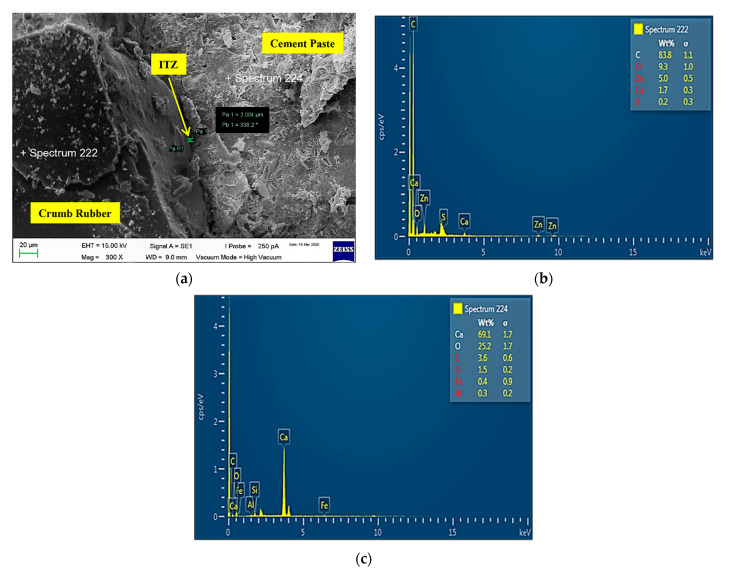
CR7.5-NS2.5 sample. (**a**) Microstructure ITZ = 2.004 µm; (**b**) EDX of crumb rubber; (**c**) EDX of cement.

Figure 17 shows the microstructure and EDX images of sample CR7.5-NS2.5. Based on the image analysis produced, ITZ created between the cement matrix and CR was 2.004 µm. Mohammed et al. [67] stated that incorporating NS into the mixture will refine the size of pores and densify the ITZ between cement matrix and CR. NS has high pozzolanic reactivity where it reacts with excess calcium hydrate (CH) and produces more C-S-H gel which fills up the pores in the cement matrix. It confirmed that NS was able to increase the density, reduce porosity and improve the bonding between CR and cement paste. At the same time, it enhances the durability as well as the strengths of cement composites. Thus, the ITZ thickness created decreases due to the physico-chemical effect of NS. According to Liu et al. [20], the microstructure of the ITZ between aggregate and the cement paste appears to be denser with the presence of NS. The improvement in the ITZ microstructure is probably due to the reaction between NS and CH which combine some water in their products and reduce the porosity of ITZ. Supported by Jaishankar [22], the pozzolanic reaction densifies the microstructures by turning CH into secondary C-S-H and produces a more homogeneous microstructure. No large pores are easily found and therefore, it is expected that the mechanical and durable properties of the samples can be improved due to this densified ITZ, which is normally the weakest part in cement composites.

3 CR7.5-NS5

**Figure 18:**
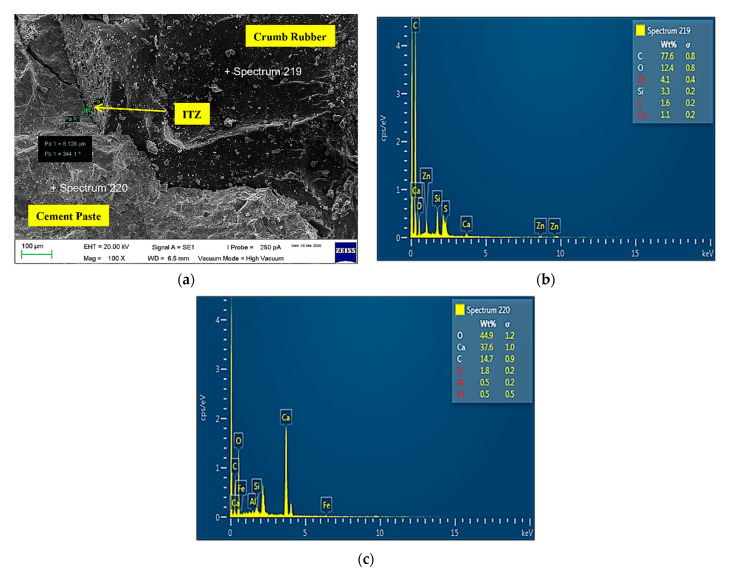
CR7.5-NS5 sample. (**a**) Microstructure ITZ = 8.128 µm; (**b**) EDX of crumb rubber; (**c**) EDX of cement.

Figure 18 shows the microstructure and EDX images for sample CR7.5-NS5. Based on the image analysis produced, ITZ created between the cement matrix and CR was 8.128 µm. Li et al. [59] stated that incorporating NS into a mixture will refine the size of the pores and densify the ITZ between cement matrix and CR. However, it is important to note that when a high percentage of NS is used, it can actually decrease the strength of the composite instead of improving it. This is due to the difficulty of NS to disperse uniformly when excessive NS is used. Hence, a weak zone is created in the form of voids; consequently, a homogenous hydrated microstructure cannot be formed. Thus, the ITZ thickness created was larger as compared to a mix design containing 2.5% of NS. Supported by Faez et al. [68], when the use of NS seems to be high in combination with the free lime produced in the hydration process, this causes the strength to decrease. The unreacted NS does not help the strength to increase and leads to defect in strength. Another reason is that NS tends to become bulky and agglomerate because of its high surface energy. Therefore, the percentage of NS which is more than the required amount results in uneven distribution, and NS appears as weak mass regions. Likewise, Mohammed et al. [69] stated that too much NS gives an adverse effect on the hardened mixture, and NS particles tend to coagulate because they exceed the limit that CH can accommodate.

#### 3.3.2. Mercury Intrusion Porosimetry

**Figure 19:**
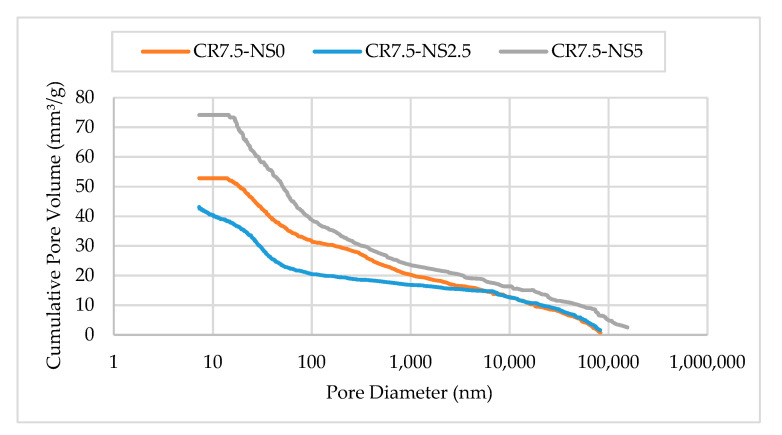
Pore size distribution of NS-CRM samples.

Mercury Intrusion Porosimetry (MIP) test was used to study the effect of physico-chemical reaction of NS on CRM. The presence of NS results in the improvement of pore structure in the hardened matrix of CRM by filling the voids of ITZ, thereby breaking the interconnectivity, and decreasing its diameter. Figure 19 illustrates the pore size distribution of samples CR7.5-NS0, CR7.5-NS2.5, and CR7.5-NS5. Based on the graph plotted, the pore diameter for CR7.5-NS0 and CR7.5-NS2.5 ranged from 7 nm to 83,000 nm whereas for CR7.5-NS5 ranged from 10 nm to 100,000 nm. Evidently, a mix design with the highest percentage of NS recorded the highest cumulative pore volume as compared to the other two mix designs. Agreed by Li et al. [70] and Du et al. [71], the higher pore volume in the pore size distribution shows that the mixture contains higher porosity and permeability, as permeability is control by large capillaries. According to Mohammed and Adamu [72], the addition of NS further reduces the total pore volumes of the mixture. The total pore volume of mixes containing NS is found to decrease compared to the one without NS. As stated by Mohammed and Azmi [73], this is due to the ability of NS to densify and improve the pore structure of the mixture up to nano-size due to the increase in the pozzolanic reaction between silicon dioxide (SiO_2_) and unreacted Ca(OH)_2_ from cement hydration products to produce more C-S-H gels. As explained by Mohammed et al. [74], the high porosity of ITZ can be lessened through physico-chemical effects, while increasing the C-S-H gel and filling up of nano-pores will result in the improvement of the ITZ and reducing pore size. The hardened specimens are interconnected, therefore, and incorporating NS helps in decreasing the total pore volume and consequently reducing the porosity.

## 4. Conclusions

The following conclusions are drawn based on the experiments and analysis results.

1. The compressive strength, flexural tensile strength, direct tensile strength, Modulus of Elasticity (ME), and Poisson’s Ratio (PR) of crumb rubber mortar containing nano-silica (NS-CRM) decreased as the percentage of crumb rubber (CR) was increased. However, an increase in drying shrinkage and porosity of NS-CRM was observed. This is attributed to the hydrophobic properties of CR which causes an increment of voids in the mixture.
2. The compressive strength, flexural tensile strength, direct tensile strength, ME, and PR of NS-CRM increased when 2.5% of nano-silica (NS) was incorporated. However, a decrease in drying shrinkage and porosity of NS-CRM was observed. This is attributed to the pozzolanic reaction of NS and its nanoparticle size.
3. The mechanical properties of NS-CRM acted contrariwise when 5% of NS was incorporated. This is attributed to the difficulty of NS to disperse uniformly when excessive NS is used. Hence, a weak zone is created in form of voids; consequently, the homogenous hydrated microstructure cannot be formed.
4. The models developed using RSM for predicting the mechanical strengths of NS-CRM were acceptable, with errors less than 5%.
5. The interfacial transition zone (ITZ) and cumulative pore volume of CRM were improved with the presence of NS. This is attributed to the physico-chemical effect of NS. However, a high percentage of NS causes the mesopores volume to increase due to agglomeration of unreacted NS which leads to excessive self-desiccation.

## Figures and Tables

**Table 1:** Chemical compositions of cementitious materials.

Chemical Compositions	Cement (%)	Nano-Silica (%)
CaO	62.85	–
SiO_2_	25.21	99.80
Al_2_O_3_	4.59	–
Fe_2_O_3_	2.99	–
MgO	1.70	–
Na_2_O	0.98	–
K_2_O	1.68	–
Loss of Ignition	2.02	6.00

**Table 2:** Physical properties of cementitious materials.

Physical Properties	Cement	Nano-Silica
Specific Gravity (kg/cm^3^)	3.15	–
Density (g/cm^3^)	3.02	0.15
Average Particle Size (nm)	13	10–25
Specific Surface Area (cm^2^/g)	0.38	100 ± 25

**Table 3:** Physical properties of fine aggregates.

Physical Properties	Sand	Crumb Rubber
Specific Gravity (kg/cm^3^)	2.65	0.95
Fineness Modulus	2.20	0.92
Water Absorption (%)	2.10	–
Moisture Content (%)	1.30	–

**Table 4:** Chemical compositions of superplasticizer.

**Chemical Composition**	Aqueous solution of modified polycarboxylates
**Appearance/Color**	Brownish liquid, clear to slightly cloudy
**pH-value**	3–5
**Total Chloride Ion Content (%)**	<0.1

**Table 5:** Constituent materials for mortar mixes.

No	Mix	Cement (kg/m^3^)	Sand (kg/m^3^)	Water (kg/m^3^)	NS (kg/m^3^)	CR (kg/m^3^)	SP (kg/m^3^)
1	CR0-NS0	0.1126	0.1688	0.0394	0.0000	0.0000	0.0198
2	CR7.5-NS0	0.1126	0.1560	0.0394	0.0000	0.0126	0.0228
3	CR15-NS0	0.1126	0.1434	0.0394	0.0000	0.0254	0.0361
4	CR0-NS2.5	0.1096	0.1688	0.0394	0.0028	0.0000	0.0217
5	CR7.5-NS2.5	0.1096	0.1560	0.0394	0.0028	0.0126	0.0257
6	CR7.5-NS2.5	0.1096	0.1560	0.0394	0.0028	0.0126	0.0255
7	CR7.5-NS2.5	0.1096	0.1560	0.0394	0.0028	0.0126	0.0260
8	CR7.5-NS2.5	0.1096	0.1560	0.0394	0.0028	0.0126	0.0250
9	CR7.5-NS2.5	0.1096	0.1560	0.0394	0.0028	0.0126	0.0263
10	CR15-NS2.5	0.1096	0.1434	0.0394	0.0028	0.0254	0.0381
11	CR0-NS5	0.1068	0.1688	0.0394	0.0056	0.0000	0.0228
12	CR7.5-NS5	0.1068	0.1560	0.0394	0.0056	0.0126	0.0283
13	CR15-NS5	0.1068	0.1434	0.0394	0.0056	0.0254	0.0400

where NS: Nano-silica, CR: Crumb rubber, SP: Superplasticizer.

**Table 6:** Test methods for mortar mixes.

Tests	Specimens	Size of Molds (mm)	Test Machines	Test Conditions	Standards
Sieve Analysis	–	–	Sieve Shaker Machines	Operate machine for 15 to 20 min	ASTM C136/C136M-14 [38]
Flow Table	–	–	Flow Table with Mold and Tamping Rod	Acceptable flow of 110 ± 5% with 25 drops in 15 s	ASTM C1437-13 [39]
Compressive Strength	Cube	50 × 50 × 50	ELE ADR 3000 Machine	Continuous load rate of 0.9 kN/s	ASTM C109/C109M-16a [40]
Flexural Tensile Strength	Prism	25 × 100 × 500	200 kN Universal Testing Machine	Continuous speed rate of 5 mm/min	ASTM C348-18 [41]
Direct Tensile Strength	Dog-bone	50 × 200 × 25	200 kN Universal Testing Machine	Gradual speed rate of 0.15 mm/s	ASTM D2936-20 [42]
Drying Shrinkage	Two-gang Prism	285 × 25 × 30	Digital Vernier Caliper	Store in room with temperature of 20 ± 2 °C	ASTM C596-18 [43]
Poisson’s Ratio	Cylinder	150 × 300	Digital Dial Gauge Compressometer– Extensometer and ELE ADR 3000 Machine	Continuous load rate of 5.3 kN/s	ASTM C469/C469M-14 [44]
Modulus of Elasticity					
SEM	Crushed sample	–	Pascal 440 EVO Machine	Crushed sample size of 1 × 1 cm	ASTM C1723-16 [45]
MIP	Crushed sample	–	EVO LS15 Machine	Crushed sample size of 1 × 1 cm	ASTM D4404-18 [46]

where SEM: Scanning Electron Microscope, MIP: Mercury Intrusion Porosimetry.

**Table 7:** Experimental design matrix and responses for NS-CRM.

No	Mix	CR (%)	NS (%)	σ_c_ (MPa)	σ_f_ (MPa)	σ_d_ (MPa)	Dry Shrinkage (%)	ME (GPa)	PR
1	CR0-NS0	0	0	61.04	0.79	2.36	0.07	28.91	0.37
2	CR7.5-NS0	7.5	0	30.31	0.54	1.52	0.08	15.79	0.19
3	CR15-NS0	15	0	19.09	0.31	1.35	0.09	9.14	0.06
4	CR0-NS2.5	0	2.5	67.77	0.90	2.40	0.06	30.44	0.44
5	CR7.5-NS2.5	7.5	2.5	35.49	0.73	1.65	0.07	16.74	0.22
6	CR7.5-NS2.5	7.5	2.5	35.45	0.78	1.63	0.07	16.01	0.25
7	CR7.5-NS2.5	7.5	2.5	36.11	0.72	1.64	0.07	17.23	0.20
8	CR7.5-NS2.5	7.5	2.5	34.90	0.72	1.66	0.07	17.08	0.19
9	CR7.5-NS2.5	7.5	2.5	35.50	0.72	1.67	0.07	16.71	0.21
10	CR15-NS2.5	15	2.5	21.10	0.50	1.52	0.08	10.23	0.10
11	CR0-NS5	0	5	54.19	0.62	2.20	0.08	28.09	0.34
12	CR7.5-NS5	7.5	5	28.06	0.40	1.43	0.09	15.07	0.17
13	CR15-NS5	15	5	16.99	0.19	1.23	0.10	7.63	0.04

where CR: Crumb rubber, NS: Nano-silica, σ_c_: Compressive strength, σ_f_: Flexural tensile strength, σ_d_: Direct tensile strength, ME: Modulus of Elasticity, PR: Poisson’s Ratio.

**Table 8:** ANOVA for developed response models.

Responses	Variables	SS	DF	µ^2^	F-Values	*p*-Values Prob > F	Significant
Compressive Strength	Model	2902.57	5	580.51	113.91	<0.0001	Yes
	A-CR	2638.45	1	2638.45	517.71	<0.0001	Yes
	B-NS	20.91	1	20.91	4.10	0.0825	No
	AB	5.64	1	5.64	1.11	0.3277	No
	A^2^	193.72	1	193.72	38.01	0.0005	Yes
	B^2^	130.54	1	130.54	25.61	0.0015	Yes
	Lack of Fit	18.61	3	6.20	1.45	0.3527	No
Flexural Tensile Strength	Model	0.52	5	0.10	138.81	<0.0001	Yes
	A-CR	0.29	1	0.29	383.96	<0.0001	Yes
	B-NS	0.032	1	0.032	42.86	0.0003	Yes
	AB	6.50 × 10^−4^	1	6.50 × 10^−4^	0.86	0.3836	No
	A^2^	6.53 × 10^−4^	1	6.53 × 10^−4^	0.87	0.3827	No
	B^2^	0.16	1	0.16	216.39	<0.0001	Yes
	Lack of Fit	2.17 × 10^−3^	3	7.23 × 10^−4^	0.93	0.5031	No
Direct Tensile Strength	Model	1.66	5	0.33	285.63	<0.0001	Yes
	A-CR	1.36	1	1.36	1172.76	<0.0001	Yes
	B-NS	0.022	1	0.022	19.15	0.0032	Yes
	AB	2.72 × 10^−4^	1	2.72 × 10^−4^	0.23	0.6427	No
	A^2^	0.26	1	0.26	228.10	<0.0001	Yes
	B^2^	0.081	1	0.081	69.74	<0.0001	Yes
	Lack of Fit	4.23 × 10^−3^	3	1.41 × 10^−3^	1.46	0.3527	No
Drying Shrinkage	Model	1.48 × 10^−3^	5	2.96 × 10^−4^	102.13	<0.0001	Yes
	A-CR	6.00 × 10^−4^	1	6.00 × 10^−4^	206.91	<0.0001	Yes
	B-NS	1.13 × 10^−4^	1	1.13 × 10^−4^	38.85	0.0004	Yes
	AB	0.00	1	0.00	0.00	1.0000	No
	A^2^	3.80 × 10^−6^	1	3.80 × 10^−6^	1.31	0.2902	No
	B^2^	6.92 × 10^−4^	1	6.92 × 10^−4^	238.60	<0.0001	Yes
	Lack of Fit	7.10 × 10^−6^	3	2.37 × 10^−6^	0.72	0.5915	No
Modulus of Elasticity	Model	641.06	5	128.21	765.52	<0.0001	Yes
	A-CR	608.79	1	608.79	3634.91	<0.0001	Yes
	B-NS	1.55	1	1.55	9.26	0.0188	Yes
	AB	0.12	1	0.12	0.71	0.4277	No
	A^2^	30.23	1	30.23	180.51	<0.0001	Yes
	B^2^	7.06	1	7.06	42.14	0.0003	Yes
	Lack of Fit	0.28	3	0.095	0.43	0.7454	No
Poisson’s Ratio	Model	0.16	5	0.032	73.58	<0.0001	Yes
	A-CR	0.15	1	0.15	345.32	<0.0001	Yes
	B-NS	8.05 × 10^−4^	1	8.05 × 10^−4^	1.84	0.2168	No
	AB	3.06 × 10^−6^	1	3.06 × 10^−6^	7.01 × 10^−3^	0.9356	No
	A^2^	4.87 × 10^−3^	1	4.87 × 10^−3^	11.14	0.0124	Yes
	B^2^	7.47 × 10^−3^	1	7.47 × 10^−3^	17.1	0.0044	Yes
	Lack of Fit	9.79 × 10^−4^	3	3.26 × 10^−4^	0.63	0.6344	No

where SS: Sum of squares, DF: degree of freedom, µ^2^: Mean square, F-values: Fisher statistical test values, *p*-values: Probability values, A: Crumb rubber, B: Nano-silica, AB: Interaction effects, A^2^ and B^2^: Second order effect.

**Table 9:** Model validation for developed response models.

Responses	SD	µ	R^2^	Adj R^2^	Pred R^2^	AP
Compressive Strength	2.26	36.93	0.99	0.98	0.93	32.27
Flexural Tensile Strength	0.03	0.61	0.99	0.98	0.96	39.83
Direct Tensile Strength	0.034	1.71	1	0.99	0.97	50.83
Drying Shrinkage	1.70 × 10^−3^	0.077	0.99	0.98	0.94	34.72
Modulus of Elasticity	0.41	17.62	1	1	0.99	80.66
Poisson’s Ratio	0.021	0.21	0.98	0.97	0.94	26.75

where SD: Standard deviation, µ: Mean, R^2^: Degree of correlation, Adj R^2^: Adjusted the degree of correlation, Pred R^2^: Predicted degree of correlation, AP: Adequate precision.

**Table 10:** Multi-objective optimization criteria.

Variables and Responses	Goals	Lower Limits	Upper Limits
Crumb Rubber (%)	In Range	0	15
Nano-Silica (%)	In Range	0	5
Compressive Strength (MPa)	Target: 20, 35, 50 MPa	16.99	67.77
Flexural Tensile Strength (MPa)	In Range	0.19	0.90
Direct Tensile Strength (MPa)	In Range	1.23	2.40
Drying Shrinkage (%)	In Range	0.06	0.10
Modulus of Elasticity (GPa)	In Range	7.63	30.44
Poisson’s Ratio	In Range	0.04	0.44

**Table 11:** NS-CRM models verification.

Mix	Validation	σ_c_ (MPa)	σ_f_ (MPa)	σ_d_ (MPa)	DS (%)	ME (GPa)	PR
CR12-NS5	Predicted	20.00	0.330	1.282	0.093	10.159	0.085
	Experimental	19.58	0.322	1.249	0.091	9.894	0.083
	Error (%)	2.10	2.42	2.57	2.68	2.61	2.58
CR8-NS3	Optimization	35.00	0.698	1.616	0.072	16.365	0.208
	Experimental	34.25	0.679	1.581	0.071	15.998	0.203
	Error (%)	2.14	2.72	2.17	2.07	2.24	2.40
CR3-NS2	Optimization	50.00	0.845	1.995	0.065	23.176	0.315
	Experimental	48.96	0.826	1.955	0.063	22.623	0.307
	Error (%)	2.08	2.25	2.01	2.63	2.39	2.54

where CR: Crumb rubber, NS: Nano-silica, σ_c_: Compressive strength, σ_f_: Flexural tensile strength, σ_d_: Direct tensile strength, DS: Drying shrinkage, ME: Modulus of Elasticity, PR: Poisson’s Ratio.

## Data Availability

The data presented in this study are available on request from the corresponding author, B.S.M. The data are not publicly available due to them containing information that could compromise the privacy of research of the first author, S.S.
